# The *Staphylococcus aureus* complex: implications for the clinical microbiology laboratory

**DOI:** 10.1128/jcm.01276-24

**Published:** 2025-06-04

**Authors:** Austin Yan, Julianne V. Kus, Nadia Sant

**Affiliations:** 1 Department of Pathology and Laboratory Medicine, Faculty of Medicine, University of Ottawa 153008 , Ottawa, Ontario, Canada; 2 Division of Microbiology, The Ottawa Hospital, Ottawa, Ontario, Canada; 3 Public Health Ontario 153300, Toronto, Ontario, Canada; 4 Department of Laboratory Medicine and Pathobiology, University of Toronto 7938, Toronto, Ontario, Canada; 5 Eastern Ontario Regional Laboratory Association 726218 , Ottawa, Ontario, Canada; 6 The Ottawa Hospital Research Institute, Ottawa, Ontario, Canada; Vanderbilt University Medical Center, Nashville, Tennessee, USA

**Keywords:** *Staphylococcus aureus*, *Staphylococcus*, diagnostics, genotypic identification, phenotypic identification

## Abstract

In the last 15 years, advances in diagnostic microbiology have enabled more detailed characterization of human bacterial pathogens. These changes have led to the description of new species within the *Staphylococcus aureus* complex, including *S. aureus* subspecies *anaerobius*, *Staphylococcus argenteus*, and *Staphylococcus schweitzeri*. In this minireview, we discuss the history, epidemiology, microbiology, genomics, and clinical significance of the *S. aureus* complex and provide a guide for laboratories to reliably detect and differentiate these novel species.

## INTRODUCTION

*Staphylococcus aureus* is a globally prevalent bacterial pathogen that causes significant human morbidity and mortality. Surveillance efforts combined with molecular and genomic advances in diagnostic microbiology have enabled detailed description and differentiation of new distinct species and subspecies that are now considered members of the *S. aureus* complex (SAC), including *Staphylococcus argenteus*, *Staphylococcus schweitzeri*, and *S. aureus* subsp. *anaerobius*. As these SAC members have been increasingly present in case reports, surveillance efforts, and microbiology guidelines, we sought to review the published literature on this topic. Here, we examine the history, epidemiology, clinical relevance, and microbiology of the *S. aureus* complex. We will also review the current methods and their limitations for identifying and differentiating SAC members, including biochemical reactions, matrix-assisted laser-desorption-ionization time of flight mass spectrometry (MALDI-ToF MS), multilocus sequence typing, polymerase chain reaction (PCR) assays, and gene sequencing.

## MEMBERS OF THE *S. AUREUS* COMPLEX

### S. aureus sensu stricto

*S. aureus* is a bacterial pathogen responsible for over 1 million deaths annually and is the leading cause of fatal bloodstream infections and skin and soft tissue infections worldwide (1, 2). *S. aureus* also causes osteomyelitis, endocarditis, pneumonia, toxic shock syndrome, and food poisoning and produces a wide variety of virulence factors and toxins that contribute to its high human mortality and morbidity (3–5). Methicillin-resistant *S. aureus* (MRSA), which confers resistance to commonly used beta-lactam antibiotics, is also among the world’s deadliest drug-resistant pathogens and continues to be a WHO high-priority bacterial pathogen as of 2024 (6).

*S. aureus sensu stricto* includes two accepted subspecies: *S. aureus* subsp. *aureus* and *S. aureus* subsp. *anaerobius*. Nearly all human *S. aureus* infections are attributed to *S. aureus* subsp. *aureus*, which is also associated with colonization and disease in a wide range of animal hosts including companion animals, ruminants, poultry, and aquatic mammals (7). In contrast, *S. aureus* subsp. *anaerobius* is a zoonotic pathogen of goats and sheep and is rarely isolated in human specimens.

### *S. aureus* subspecies *aureus*

*S. aureus* subspecies *aureus* (hereby referred to as *S. aureus*) colonizes several human body sites serving as a reservoir for infection, particularly in the nasal cavity where it is persistently or intermittently present in up to 50% of individuals (8). *S. aureus* is also frequently isolated from skin, throat, gastrointestinal, and vaginal samples (8). *S. aureus* is an active topic of infectious disease research, including several ongoing clinical trials on optimal treatment of *S. aureus* bacteremia, novel antimicrobials, and vaccine development (9–11).

Given its high prevalence and clinical significance, *S. aureus* is one of the most commonly encountered bacteria in the clinical microbiology laboratory. Like other staphylococci, *S. aureus* is catalase-positive, facultatively anaerobic, gram-positive cocci bacteria that appear as “grape-like” clusters on direct staining. On blood agar, *S. aureus* colonies are golden, yellow, or cream-color colonies that are beta-hemolytic and have a positive coagulase test (12). Combined, these features were previously sufficient for identifying *S. aureus*, although they are unable to differentiate *S. aureus* from recently delineated SAC members that share most of these characteristics with key exceptions in aerotolerance and pigmentation detailed below. Modern tools including MALDI-ToF MS or nucleic-acid amplification tests are commonly used to identify *S. aureus* in laboratories today (13).

Other staphylococci that are coagulase-positive include *S. intermedius*, *S. coagulans* (formerly *S. schleiferi* subsp. *coagulans*), *S. hycius*, and *S. lutrae* (14). These coagulase-positive staphylococci, outside of SAC members, are infrequently associated with human disease and readily distinguished from SAC based on colony appearance, carbohydrate fermentation (mannitol, maltose), the Voges-Proskauer reaction, and MALDI-ToF MS (14).

### *S. aureus* subspecies *anaerobius*

*S. aureus* subsp. *anaerobius* is a zoonotic, abscess-forming, microaerophilic bacterium best known for causing “Morel’s disease” in sheep and goats (15). First identified in the 1950s as *Micrococcus abscedens ovis*, it was reclassified as *S. aureus* subsp. *anaerobius* in the 1980s based on similar biochemical reactions, metabolism, cell wall composition, and DNA-DNA hybridization profiles to *S. aureus* and distinguished by its need for anaerobic or microaerophilic conditions (15). Unlike other staphylococci, this subspecies is catalase-negative due to an impaired catalase gene (16). Human infections with this subspecies are exceedingly rare, including a case report of recurrent abscesses in a patient with sheep exposure with an isolate biochemically consistent with *S. aureus* subsp. *anaerobius* (17). A second report described septicemia with an anaerobic *S. aureus* isolate; however, weak catalase activity and sequencing efforts argue against an identification of *S. aureus* subsp. *anaerobius* (18). This subspecies remains understudied with only one published genome study which showed 99% 16S rRNA gene nucleotide sequence similarity and approximately 98% nucleotide and amino acid identity across its genome with *S. aureus* subspecies *aureus* (16, 19). Subspecies-level identification is not routinely performed in microbiology laboratories, and thus, its true epidemiology and clinical significance in humans remain unclear.

### S. argenteus

*S. argenteus* was first identified as a locally predominant clonal complex of *S. aureus* (CC75) in northern Australia in a survey of MRSA isolates (20). CC75 was later found globally, at times through retrospective examination, suggesting its widespread and even historic distribution (20–28). Unlike *S. aureus*, the CC75 isolates lacked the golden pigment staphyloxanthin, resulting in grey-white strains that were increasingly reported as a “phylogenetically distinct *S. aureus* lineage” in the following years (29–32). *S. argenteus* or “silver staph” was formally described and proposed as a new species in 2013; the type strain, isolated from the blood of an Indigenous Australian woman, was described in 2015 (30, 33, 34).

Like *S. aureus*, *S. argenteus* causes a wide range of infections, including skin and soft tissue infections (30, 35, 36); osteomyelitis and prosthetic joint infections (37–39); bloodstream infections and endocarditis (40–44); and staphylococcal foodborne illness outbreaks (45–48). *S. argenteus* isolates have also been identified in gorillas, macaques, dairy cattle, fruit bats, rabbits, and dogs (49–54).

The prevalence of *S. argenteus* relative to *S. aureus* varies by study and region and is unknown and likely underreported in most settings. In Northern Australia (*n* = 1,243) and Fiji (*n* = 122), *S. argenteus* represented 8%–10% of SAC isolates and was often associated with community-acquired impetigo but rarely with sepsis (33, 55). In contrast, *S. argenteus* represented 11.9%–18.6% of SAC bacteremia in Thailand and Taiwan (26, 56). However, larger studies (>1,000 SAC isolates) in Japan and Europe suggest that *S. argenteus* makes up no more than 0.6% of SAC isolates (24, 57–59). These differences suggest that some areas (i.e., Southeast Asia, Northern Australia, the Amazon) may be “hot spots” for *S. argenteus* (12, 60). *S. argenteus* is also associated with asymptomatic colonization, with studies in the UK and Japan reporting *S. argenteus* carriage rates of 0.09%–2.3%, compared with 41.0%–47.4% by *S. aureus* (61–63). Surveillance efforts also demonstrate long-term colonization, loss and acquisition of new strains, and household transmission of *S. argenteus* (24, 64).

Few studies directly compare the clinical impact of *S. aureus* and *S. argenteus* infections. Early reports suggested that *S. argenteus* infections were less severe, particularly in Northern Australia where most infections were community-associated, superficial skin infections (30, 33, 35). Animal models also showed lower disease severity, alongside reports of reduced drug resistance and fewer virulence factors among clinical *S. argenteus* isolates (50, 56, 65). In contrast, other studies in Asia have shown similar or increased mortality, including a single-site retrospective observational study in Taiwan that found *S. argenteus* to have a higher 90-day mortality rate than methicillin-susceptible *S. aureus* (56, 60). The ability to compare studies is limited by differences in diagnostic techniques in identifying *S. argenteus*, which has evolved over the past decade. Moreover, regional variability in sequence types that are associated with antibiotic resistance and specific virulence factors should also be considered when studying clinical differences between SAC members.

### S. schweitzeri

*S. schweitzeri* is mainly found in African wildlife and was formally described alongside *S. argenteus* in 2015 with a distinct phylogeny and ecological niche (34). Unlike *S. argenteus*, *S. schweitzeri* shares the classic yellow or golden appearance of *S. aureus* without any other distinctive features on colony morphology. *S. schweitzeri* is named after Hôpital Albert Schweitzer in Lambaréné, Gabon, with its initial representative strains obtained from non-human primates in Gabon and Côte d'Ivoire (34). *S. schweitzeri* has since been isolated primarily in non-human primates and bats, although strains have been found to colonize the human nasopharynx in individuals with animal exposures and on fomites such as banknotes and computer keyboards (66, 67). Unlike the globally distributed *S. aureus* and *S. argenteus*, all isolates of *S. schweitzeri* to date have been reported in Sub-Saharan Africa (12, 67).

There have been no reports of *S. schweitzeri* human infections, although genomic and *in vitro* analyses demonstrate the presence of most *S. aureus*-related virulence factors and capacity for cytotoxic effect in human cell lines (67, 68). One *in vitro* study showed that *S. schweitzeri* grows better at 34°C and 40°C and worse at 37°C when compared with *S. aureus*, which has been hypothesized to demonstrate adaptation to non-human hosts and explain its association with zoonotic infections (67).

### *S. singaporensis* and *S. roterodami*

There are only two other members of the *S. aureus* complex that have been published in the International Journal of Systematic and Evolutionary Microbiology, both introduced in 2021 and differentiated from other SAC members by genomic analysis. A non-pigmented SAC isolate was identified in a traveler in a medical center in Rotterdam, Netherlands, who had recently returned from Bali, Indonesia. This isolate was initially identified as *S. argenteus* by MALDI-ToF MS but was proposed as *S. roterodami* after genomic analysis revealed significant divergence from other SAC members, including an average nucleotide identity (ANI) of 88.0% with *S. aureus* and 93.3% with *S. argenteus* (95% is often used as a threshold for new species) (19). *S. singaporensis* was identified as a genetically distinct clade (ANI <94%) in a survey of human non-*S*. *aureus* SAC isolates from Singapore (69). All *S. singaporensis* isolates contained the staphyloxanthin biosynthetic cluster with yellow-pigmented colonies (69). Subsequent core genomic analysis has suggested that *S. roterodami* and *S. singaporensis* should be regarded as a single species (70). Additional isolates may be required before these two species are more widely recognized within the SAC.

## PHYLOGENY, VIRULENCE, AND ANTIMICROBIAL RESISTANCE OF THE *S. AUREUS* COMPLEX

### Comparative genomics

Most SAC genomes primarily consist of circular chromosomes of 2.65–2.9 Mb with a low guanine-cytosine content of approximately 32% (19, 69, 71). *S. aureus* subsp. *anaerobius* has a slightly smaller genome of 2.60 Mb due to gene loss, suggesting the emergence of a clone adapted for animal infection (16, 72). Accessory gene content, including several virulence factors and antimicrobial resistance genes, is present across genomic and pathogenicity islands, prophages, staphylococcal cassette chromosomes (SCC), plasmids, and transposons (73).

Phylogenetic analysis enabled by advancing sequencing technologies in the early 2010s led to the designation of *S. argenteus* and *S. schweitzeri* as distinct species. Core genome analysis revealed a 7-fold increase in nucleotide divergence between *S. argenteus* and *S. aureus sensu stricto*, where these isolates had a ~90% average nucleotide identity (ANI, a measure of genomic stability) compared with ~2% ANI between *S. aureus* isolates (30). At a 90% homology level, one-third of the gene pool was shared between *S. argenteus* and *S. aureus*, whereas only 4% of genes were shared at a 100% homology level (74). Other factors demonstrating divergence from the *S. aureus* lineage include loss of the staphyloxanthin biosynthetic cluster and independent insertions and recombination of prophages (30). *S. schweitzeri* is also phylogenetically distinct with less than 90% ANI compared with both *S. aureus sensu stricto* and *S. argenteus* (34).

The ANI is higher between *S. argenteus* and *S. schweitzeri* (92.0%–92.6%) than *S. argenteus* and *S. aureus* (87.4%–89.0%) (34, 75, 76) (Table 1). Thus, *S. argenteus* and *S. schweitzeri* likely share a more recent common ancestor (77). One study suggested that if an ANI threshold of 95% is used, *S. roterodami* and *S. singaporensis* would be clustered together, whereas a threshold of 90% would result in only two SAC clusters: *S. aureus* as one species and all other SAC members as the second (70).

**TABLE 1:** Key members of the *S. aureus* complex*^e^*

Species	*S. aureus*	*S. argenteus*	*S. schweitzeri*
Subspecies	*aureus*, *anaerobius*	Not described	Not described
Predominant host	Human	Human	Wildlife
Genome size (based on complete genome assemblies)	2.665-3.219 Mb^*c*^ 2.604 Mb^*d*^	2.698-2.873 Mb	2.785 Mb
bANI^*a*^ (vs. *S. aureus*)	98.78% ± 1.92%	89.79% ± 4.63%	90.92% ± 4.77%
bANI^*a*^ (vs. *S. argenteus*)	89.79% ± 4.63%	99.60% ± 0.85%	94.08% ± 3.69%
Available complete genome assemblies	2,224	16	1
Available genomes^*b*^	102,267	432	9

^*a*^ bANI (BLAST ANI) was calculated using core gene comparisons among 51 SAC genomes by D. F. Zhang et al. (78).

^*b*^ In contrast to complete assemblies, available genomes include chromosomes, scaffolds, and contigs available on NCBI datasets.

^*c*^ Genome sizes outside this range have been reported but have been flagged for abnormal genome size or lack of completeness.

^*d*^ Genome size of *S. aureus* subsp. *anaerobius*.

^*e*^ Genome sizes and available sequences reflect data available on NCBI Datasets up until 2024.

The *S. argenteus* mutation rate is estimated at 4.66 mutations/genome per year, similar to *S. aureus*, which would suggest a split from a common ancestor around 41,000 years ago (25, 57). *S. argenteus* is thus proposed to have evolved as an ancient, insulated *S. aureus* strain, with the more recent horizontal acquisition of select accessory genes (30, 32, 79). For example, although some enterotoxin-containing genomic islands are 92% identical between *S. argenteus* and *S. aureus*, several antibiotic resistance genes including the methicillin resistance conferring SCC*mec* and other virulence factors are nearly 100% identical (25, 30, 57). Similar analysis in other SAC members is not yet available.

### Virulence factors of *S. aureus* and *S. argenteus*

*S. aureus* is known for a broad range of virulence factors, many of which are shared with *S. argenteus*. Both species carry staphylocoagulase, protein A, alpha-hemolysin, staphylokinase, and several enterotoxins that are genetically distinct, suggesting that they evolved alongside the rest of the *S. argenteus* genome rather than via recent horizontal acquisition (24, 71, 80). The *S. aureus*-specific staphyloxanthin may contribute to oxidation resistance and reduced neutrophil killing (30, 33). Mouse models of mutant staphyloxanthin-producing *S. argenteus* strains, however, do not show a clear link between virulence and staphyloxanthin production (33, 77, 81).

Toxic shock syndrome toxin-1 (TSST-1), exfoliative toxin A (which causes staphylococcal scalded skin syndrome), and leukocidins including LukED and Panton-Valentine leukocidin (PVL) are rarely identified in *S. argenteus* (47, 56, 71, 74, 76, 82–88). Some strains carry just one of the two leukocidin-encoding genes with unclear clinical significance (56, 74). The prevalent *S. aureus* enterotoxin SEA has not been detected in any *S. argenteus* genomes, whereas SEB-producing *S. argenteus* has been identified in foodborne outbreaks but appears to be rare overall (47, 74, 89). Most *S. argenteus* isolates harbor enterotoxin gene cluster (*egc*) type 6, with some sequence types also highly associated with staphylococcal enterotoxin-like genes *sel26* and *sel27* (30, 47, 90, 91).

Both *S. argenteus* and *S. schweitzeri* demonstrated high *in vitro* cytotoxicity to human cancer cells due to increased expression of *hla*, which encodes the alpha-hemolysin Hla (68). *S. argenteus* isolates that caused persistent hip joint infections were also found to be able to produce small colony variants, bone cell invasion, and biofilm formation that could promote persistent infection, with possible induction by antimicrobial therapy (39, 92). Although no novel *S. argenteus*-specific virulence factors have been identified, most studies rely on comparisons with known *S. aureus* genes. Further studies dedicated to each SAC member may be required to understand if there are unique aspects to species-specific virulence and pathogenicity.

### Antibiotic resistance in *S. aureus* and *S. argenteus*

Antimicrobial resistance, particularly methicillin resistance, is a major contributor to the morbidity and mortality associated with SAC infections, with several implications for treatment and infection control. Staphylococcal methicillin resistance is primarily conferred by SCC*mec*, a mobile cassette that includes *mecA* or *mecC,* which encode an alternative penicillin-binding protein target, enabling resistance to most beta-lactam antibiotics (71). The high similarity of SCC*mec* elements between MRSA and methicillin-resistant *S. argenteus* (MRSArg) suggests recent horizontal transfer, with all reported MRSArg isolates containing a type IV SCC*mec* element (30, 71, 76, 78).

Generally, *S. argenteus* isolates are less likely to be methicillin-resistant or multidrug-resistant in comparison to *S. aureus* (48, 65, 85, 93). MRSArg is relatively uncommon in most regions, including East and Southeast Asia, Europe, and North America (24, 25, 76, 93). However, *S. argenteus* accounted for 68% of methicillin-resistant SAC infections compared with 8% of methicillin-susceptible isolates in Northern Australia, suggesting that in select regions, MRSArg may be clonally dominant (33). MRSArg isolates also tend to carry resistance genes for penicillin (*blaZ*) and trimethoprim (*dfrA*) (71). Among methicillin-susceptible SAC isolates, transposon-associated and plasmid-borne *blaZ* may be more prevalent in *S. argenteus* compared with *S. aureus,* although penicillin is not a common treatment choice for staphylococcal infections (47, 62, 65, 76, 84, 85, 94). The prevalence of resistance genes is closely associated with sequence type (62, 71, 74).

Like *S. aureus*, rare cases of daptomycin-resistant, vancomycin-intermediate, and/or mupirocin-resistant strains of *S. argenteus* have been reported (75, 95–97). A chromosomal fosfomycin resistance gene (*fosB*), lincosamide nucleotidyl transferases (*lnuA*), trimethoprim-resistance traits (*dfrB*, *dfrG*), and various efflux pumps have also been detected in *S. argenteus* but do not appear to reliably confer phenotypic resistance (53, 56, 64, 71, 76).

Given the evidence demonstrating the potential severity of infections due to *S. argenteus*, the Clinical & Laboratory Standards Institute (CLSI) and the European Committee on Antimicrobial Susceptibility Testing (EUCAST) recommend applying *S. aureus* testing guidelines and breakpoints for *S. argenteus* “without caveats,” instead of the non-*S*. *aureus* staphylococcal breakpoints (98–100). The use of non-*S*. *aureus* staphylococcal breakpoints for *S. argenteus* may result in overcalling methicillin resistance: although *S. aureus* breakpoints correctly identified three of 21 *S*. *argenteus* isolates that were *mecA* PCR positive, an additional four *mecA* PCR-negative isolates were called methicillin-resistant using non*-S. aureus* staphylococcal breakpoints (76). Neither the CLSI nor EUCAST currently offer specific AST recommendations for *S. schweitzeri*, with the CLSI noting human infections with this organism “have yet to be reported” (98, 99). The most recent CLSI guidelines have also added citations for *S. roterodami* and *S. singaporensis* but indicate that AST methods have not yet been evaluated for these species (98). The article describing *S. roterodami* applied *S. aureus* EUCAST breakpoints and reported concordance between phenotypic testing and genomic predictions, including methicillin susceptibility and the absence of *mecA* (19).

Because methicillin resistance in *S. argenteus* is also mediated by the production of penicillin-binding protein 2 a (PBP2a) through SCC*mec*, methicillin resistance testing by *mecA* PCR or PBP2a detection would be expected to detect MRSArg and should be applied to *S. argenteus* (101, 102). Although not formally evaluated by CLSI, the use of these methicillin resistance detection methods across staphylococci including non-SAC species suggests applicability to other SAC members beyond *S. aureus* and *S. argenteus*, with no reports of novel methicillin resistance mechanisms

## IDENTIFICATION OF *S. AUREUS* COMPLEX MEMBERS

All SAC members are gram-positive cocci that are catalase-positive, tube- and slide coagulase-positive, and beta-hemolytic on blood agar (12, 34). Despite advances in diagnostic microbiology, differentiating SAC members can still pose challenges for modern clinical laboratories. Here, we highlight several phenotypic and genotypic features to assist with the laboratory identification of SAC members, with a focus on the human pathogens *S. aureus* and *S. argenteus*. This section is further summarized in Tables 2 and 3.

**TABLE 2:** Select methods for differentiating *S. aureus* complex members^*b*^

Test	Rationale	Limitations/notes	ESCMID rating^*a*^	Key studies
*Lack of pigmentation (colony morphology)	Absence of yellow pigment due to a lack of production of staphyloxanthin by *S. argenteus*	Low specificity (*S. aureus* can lose staphyloxanthin production); subjective	Uncertain	C. Holt et al. (30); J. Zhang et al. (81)
**Biochemical testing**	Commonly used laboratory assays that identify phenotypic differences between species	No definitive indicator	Not definitively	W. Ng et al. (31), J. C. Hsu et al. (93)
Catalase	Identifies *S. aureus* subsp. *anaerobius*	Rare cases of catalase-negative *S. aureus* subsp. *aureus*	No comment	
Obligate anaerobe	Identifies *S. aureus* subsp. *anaerobius*	Rare cases of obligately anaerobic *S. aureus* subsp. *aureus*	No comment	
Urease	56-65% / 0-8.4% for *S. argenteus* / *S. aureus*	Low specificity	No comment	See above
N-acetyl-d- glucosamine	2.5-28% / 40-98% for *S. argenteus* / *S. aureus*	Low specificity	No comment	See above
**Peptidoglycan composition**	*S. argenteus* produces a distinct type of peptidoglycan compared to *S. aureus*	Not performed in most laboratories; *S. argenteus* and *S. schweitzeri* have the same peptidoglycan	Possible	Y. C. Tong et al. (34)
*Matrix-assisted laser desorption/ionization time-of-flight mass spectrometry	Commonly used laboratory test that generates a proteomic profile that can be matched to a spectra database	Database may lack non *S. aureus* SAC spectra; may lack resolution to differentiate non-*S*. *aureus* SAC members	Possible	Y. Chen et al. (26)
*Multilocus sequence typing	Sequencing of a set of housekeeping genes to determine “sequence type” grouped into “clonal clusters”; often seen as the standard approach	Not routinely performed; methods may be modified which limits direct comparison between studies; being replaced by whole genome sequencing	Indicative	Becker et al. (12), M. McDonald et al. (20)
**Multilocus variable-number tandem-repeat analysis**	Sequence typing technique where non-typeable *S. aureus* strains may be *S. argenteus*	Not routinely performed; moderate specificity (84% *S*. *argenteus*, 9% *S*. *aureus*, 6% non-SAC bacteria)	No comment	Witteveen et al. (24)
**Pulsed-field gel electrophoresis**	DNA fingerprinting technique for strain-level differentiation often used for outbreak investigations	Primarily used only as a comparative tool; often paired with MLST; not used for identification	No comment	Gupta et al. (103)
**Polymerase chain reaction assays**				
*spa* (staphylococcal protein A gene)	Common *S. aureus* genotyping target with a variable-number tandem repeat region and publicly available databases	Requires Sanger sequencing; may benefit from *S. argenteus* specific primers; may be discordant with other typing methods	Probably indicative (unstudied)	Schuster et al. (50)
*nuc (nuclease gene)	RT-PCR target often used for *S. aureus* detection but may not be detected for some *S. argenteus* isolates	*S. argenteus* detection is variable based on primer set	Possible	Y. C. Tong et al. (34), A. Eshaghi et al. (76)
*NRPS* (non-ribosomal peptide synthetase gene)	*NRPS* has a 180 bp indel specific to *S. aureus*, enabling rapid differentiation based on PCR-product size	Does not differentiate *S. argenteus* and *S. schweitzeri*	No comment	F. Zhang et al. (104); C. Akoua-Koffi et al. (105)
*crtOPQMN* (staphyloxanthin biosynthesis operon)	The genes that encode staphyloxanthin production are absent in *S. argenteus*. Targets include the entire operon or *crtM*	Not specific, as *S. aureus* may have a defunct/absent *crt* operon.	No comment	C. Holt et al. (30); B. F. Rossi et al. (106)
**Microarray**	Genotyping method that can include hundreds of targets, including virulence factors; commercial systems available	Not commonly used; MLST is more cost-effective; being replaced by WGS	No comment	Monecke et al. (94)
*Whole genome sequencing	Genomic analysis can enable *in silico* (whole genome) MLST approaches and the detection of many targets described above.	High cost (decreasing); requires bioinformatic analysis; difficulty with assembling repetitive regions (i.e. *spa*)	Possible	C. Holt et al. (30)

^*a*^ ESCMID rating refers to the utility of each test as classified in Table 2 (Utility of diagnostic approaches to differentiate within the *S. aureus* complex) of Becker et al. (2019), an ESCMID position paper on the implication of SAC species.

^*b*^ The most commonly used methods for most clinical microbiology laboratories are marked with an asterisk.

**TABLE 3:** Select phenotypic and genotypic test characteristics of *S. aureus* complex members^*b*^

Test	*S. aureus* subspecies *aureus*	*S. aureus* subspecies *anaerobius*	*S. argenteus*	*S. schweitzeri*
Catalase	+	–	+	+
Coagulase	+	+	+	+
Urease	– (<10%)	ND	V	–
Obligate anaerobe	–	+	–	–
Golden pigment (staphyloxanthin production), *crtM* gene PCR, or *crtOPQMN* operon PCR	+ (>90%)	+	–	+
*S. aureus* nuclease gene PCR^*a*^	+	+	V	–
*NRPS* gene PCR	160 bp amplicon	160 bp amplicon	340 bp amplicon	340 bp amplicon

^*a*^ All SAC members have a nuclease gene; however, assays designed to identify *S. aureus* nuclease variably amplify *S. argenteus* nuclease and usually do not detect *S. schweitzeri* nuclease*.*

^*b*^ +, positive; –, negative; V, variable; ND, no data.

### Pigmentation

*S. argenteus* lacks the staphyloxanthin pigment that gives *S. aureus* and *S. schweitzeri* their yellow or golden color, leading to a “white,” “brilliant white,” “creamy-white,” or “silver” appearance (thus the name “argenteus”) (30, 34, 107). However, up to 10% of *S. aureus* isolates are non-pigmented, and thus, in most populations, the absence of pigmentation has low specificity for *S. argenteus* but may have utility as an initial screen (81). *S. argenteus* represented only 4%–7% of white-colony SAC in two studies (59, 104). Colony morphology and color can also be subjective, with some reports describing *S. argenteus* as “yellow-gray” (32). Pigment production may be more visible on chocolate agar after a 48 h incubation or on salt egg yolk agar (30, 34, 65).

### Biochemical phenotyping

Biochemical profiling cannot definitively discriminate between any SAC members (19, 31, 32, 34, 69). *S. argenteus* is more likely to be urease-positive (56% vs 0% for *S. aureus*) and less likely to test positive for N-acetyl-d-glucosamine (28% vs 98% for *S. aureus*) (34); however, urease positivity can also occur in *S. aureus* (30, 93). *S. argenteus* may also be more likely to utilize trehalose (30).

*S. aureus* subsp. *anaerobius* can theoretically be differentiated by a negative catalase reaction and requirement for anaerobic or microaerophilic conditions. These characteristics likely limit its isolation and accurate identification, although this subspecies is rarely relevant for human samples. Further investigation of catalase-negative or anaerobic *S. aureus* isolates from human hosts typically reveals rare variants of *S. aureus* subsp. *aureus* (18, 108).

### Chemotaxonomy

Analysis of the constitutive elements of the bacterial cell such as fatty acids, isoprenoid quinones, and peptidoglycans has been used to identify unique attributes of SAC members (34). Although fatty acid and respiratory quinones profiles were similar between all members, *S. argenteus* and *S. schweitzeri* both produce a peptidoglycan type (A11.8: l-Lys–l-Ala–Gly_4-5_) that differs from *S. aureus* (A11.2: l-Lys–Gly_4-5_) (34). Chemotaxonomic methods, however, are not readily accessible to most clinical microbiology laboratories.

### MALDI-ToF MS

The addition of *S. argenteus* to some commercial databases as early as 2018 (i.e., Bruker BioTyper RUO) has led to its increased awareness and reporting in case reports and series (38, 49, 57, 64, 76, 109). However, due to variations in MALDI-ToF MS databases, the absence of *S. argenteus* spectra has led to its misidentification as *S. aureus* as recently as 2023 (37, 75, 102, 107, 110). *S. argenteus* continues to be absent from select commercial databases including the US Food and Drug Administration-cleared VITEK MS V3.2 database and Bruker MALDI Biotyper CA reference library (Claim 6), and although these are always being updated, it is prudent for laboratories to understand the potential limitations of their systems (111, 112).

It is important to note that although *S. aureus* can be readily differentiated from *S. argenteus* and *S. schweitzeri* by MALDI-ToF MS, the converse is not always straightforward (76). When *S. argenteus* is subjected to MALDI-ToF MS testing, it is often the case that other SAC members also have high identification scores (≥ 2.0 (Bruker)), suggesting that MALDI-ToF MS may not always provide sufficient resolution to differentiate SAC members (76). Chemical extraction methods, including formic acid extraction, may assist with *S. argenteus* identification (26, 64). The inclusion of all SAC members in MALDI-ToF MS databases improved the identification of species-specific spectra, and regional validation to account for local sequence types will enable laboratories to more quickly and reliably identify SAC members.

### Genotypic identification and profiling of SAC members

#### Multilocus sequence typing (MLST)

MLST involves sequencing a series of housekeeping genes to define sequence types that can be further clustered into clonal complexes, where specific sequence types can be associated with each SAC member (20). PubMLST includes a widely adopted, seven-gene MLST scheme for *S. aureus* that has been applied to other SAC members, with over 9,400 profiles reported in 2024 (113). MLST profiling has revealed at least 60 sequence types associated with *S. argenteus* and 30 sequence types associated with *S. schweitzeri* (12). The epidemiology of sequence types varies regionally and is closely related to specific virulence factors and antibiotic resistance genes and can thus be used to track the spread of MRSA and MRSArg. MLST was the only method listed in the European Society of Clinical Microbiology and Infectious Diseases position paper as “indicative” for SAC species identification and has been the most common technique used to survey *S. argenteus* to date (12).

#### Multilocus variable-number tandem-repeat analysis (MLVA) and pulsed-field gel electrophoresis (PFGE)

MLVA shares similarity to MLST; however, it utilizes universally present tandem-repeat regions for strain typing instead of housekeeping genes. PFGE employs a restriction enzyme digest of genomic DNA to generate a strain-specific “DNA fingerprint.” Neither method is routinely performed for SAC member identification but has strain-level discriminatory power that is typically employed for epidemiology studies in outbreak settings. Notably, 54 of 64 (84%) non-typable isolates in a large MLVA-based, MRSA surveillance program were subsequently identified as *S. argenteus* (24). PGFE has been used for *S. argenteus* identification but is always paired with MLST (93, 103, 114).

#### Microarray

Microarray systems incorporate hundreds of sequence targets to enable bacterial identification (94). Multiple commercial assays have been developed for staphylococci including the StaphylType DNA microarray and the INTER-ARRAY Genotyping Kit, which can provide sequence type assignment and staphyloxanthin gene detection and thus can be used to differentiate SAC members (32, 37, 39, 61, 115).

#### Polymerase chain reaction (PCR) assays

Several gene targets for *S. argenteus* have been proposed as an alternative or complementary technique to MLST, although no commercial assay exists for SAC identification (12). Use of these targets may involve gene detection, amplicon size assessment, or gene sequencing. Notably, all SAC members share highly similar (>99%) or identical 16S rRNA gene homology, which thus cannot provide sufficient species-level resolution (37, 76). The genes *spa, nuc*, *coa*, *sau*, and *rpoB* are routinely used for *S. aureus* detection or typing and have been applied to *S. argenteus* and *S. schweitzeri* with varying success, depending on primer design and placement (41, 59, 81, 104). Clinical strains found to be “non-typeable” are often subsequently identified as *S. argenteus*, although these isolates could also represent other SAC members (34, 102, 116).

The staphylococcal protein A gene (*spa*) contains a polymorphic variable-number tandem repeat, which is the most used single PCR target for *S. aureus* sequence typing (12, 76). *spa* typing often requires dedicated Sanger sequencing even when whole genome sequencing is available due to the difficulty of assembling repetitive sequences (76). *S. argenteus*-specific *spa* primers have also been developed (50, 116). Although *spa* typing usually corresponds with MLST schemes, some *spa* types associated with *S. argenteus* were later found to be *S. aureus* based on further sequencing efforts, suggesting that *spa* typing may not be a sufficiently discriminatory target for SAC members (59).

The gene *nuc* or *nucA*, which codes for nuclease, is commonly used for the detection of *S. aureus* (76). *S. argenteus* and *S. schweitzeri* also possess the *nuc* gene; however, due to species-specific sequence differences, these assays may selectively identify *S. aureus* only or amplify the *nuc* gene from both *S. aureus* and *S. argenteus* (despite mismatches) but not from *S. schweitzeri* (12, 34, 76, 102). Nuclease gene identity between *S. aureus* and *S. argenteus* has been reported to be between 83% and 87%, with *S. aureus* encoding a longer *nuc* gene of 687 bp compared with 670 bp (41, 74). Probes that target *nuc* have thus been specifically designed to include, distinguish, or omit *S. argenteus* from MRSA screening assays (117). The possibility of *S. argenteus* being either “*nuc*-positive” or “*nuc*-negative” highlights the impact of primer design on epidemiological studies. Meanwhile, *S. schweitzeri* has a more divergent nuclease gene, sometimes referred to as *nucM*, with 78.1%–80.4% identity compared with *S. aureus nuc* (105, 118). There are no reports of *S. schweitzeri* being identified by *S. aureus nuc* assays.

The non-ribosomal peptide synthetase (*NRPS*) gene has been used to identify *S. argenteus*, targeting an indel region where *S. aureus* has a 60-amino acid deletion (104). Because the NRPS amplicon is 160 bp for *S. aureus* but 340 bp for both *S. argenteus* and *S. schweitzeri*, *S. aureus* can be excluded based on amplicon size alone (105). Given the low incidence of *S. schweitzeri* in human samples, many studies use the presence of a 340 bp amplicon as presumptive or positive identification for *S. argenteus* (104, 105).

The absence of staphyloxanthin biosynthesis genes in the *crt* cluster (primarily its first gene *crtM,* which encodes dehydrosqualene synthase) has been used to screen for *S. argenteus* isolates (26, 29, 30, 57). Primers may also target the operon-deficient region as a marker for *S. argenteus* (57). However, given that non-pigmented *S. aureus* may also lack *crt* genes, these features are not specific to *S. argenteus* (106).

Many of the above PCR targets may be combined to develop an algorithm for SAC member identification: J.H.K. Chen et al. (41) employed a panel of *nuc/sau*, *mecA*, and *coa* tests, whereas C. Akoua-Koffi et al. (105) proposed a two-step algorithm using *NRPS* product size and *nuc* detection. Robust testing algorithms should not only consider *S. aureus* and *S. argenteus* but also the possibility of all SAC members.

#### Whole genome sequencing (WGS)

Whole genome sequencing, primarily used in research settings and large reference laboratories, can enable SAC member identification, *in silico* MLST and core gene analysis, virulence factor detection, and antibiotic resistance prediction (24, 50, 74, 83, 102, 116). The specific genes or genome similarity used to label a strain as *S. argenteus* varies greatly between studies, highlighting the importance of regular quality control efforts and the standardization of interpretive criteria and bioinformatic pipelines (119).

### Reporting of *S. aureus* complex members

Given that clinical differences between *S. aureus* and *S. argenteus* remain an active area of research and that laboratories may not always reliably identify SAC members, there is ongoing debate regarding the distinct reporting of *S. argenteus*. The 2019 ESCMID position paper considers the need to routinely differentiate SAC members as “currently questionable” and that if *S. argenteus* is reported, it should be noted as a SAC member to avoid misinterpretation and underestimation of its pathogenicity (12). The CLSI also recommends that for laboratories that can reliably identify the SAC to species level, *S. argenteus* should be reported as “*S. aureus* complex (*S. argenteus*)” (100). Concerns for clinical confusion were highlighted in a case series, where only one of four patients with *S. argenteus* infection received appropriate therapy, suggesting possible misinterpretation (unawareness) as a contaminant coagulase-negative *Staphylococcus* (58). These recommendations should extend to other SAC members, whereas methicillin-resistant SAC members should also follow the same infection prevention and control protocols as MRSA (12).

## FUTURE STEPS

As methods become increasingly available to distinguish between SAC members, greater awareness of the SAC is required among microbiologists, laboratory personnel, and clinicians. For surveillance purposes and to improve our understanding of the clinical implications of each SAC member, species-level identification should be encouraged for suitably equipped laboratories (12, 76). Microbiologists should be aware of the limitations of current *S. aureus* identification systems and screening methods that could preclude the detection of other SAC species. Some *S. aureus* surveillance efforts have also been recently modified to specifically include *S. argenteus* (24).

Research on the newer SAC members remains limited by significant gaps and biases. Although larger epidemiological studies have been performed in Australia, east and southeast Asia, and Europe, similar data are lacking in the Americas, Africa, and the Middle East. About half of the strains featured in both large pangenome analyses of *S. argenteus* are from Thailand alone (71, 79). Given that many non-*S*. *aureus* SAC isolates were identified through MRSA surveillance efforts, methicillin-susceptible isolates may also be under-represented. Due to the challenges and advances in differentiating SAC members, reports between earlier and more recent surveys may not be directly comparable in the absence of a “gold standard.” Furthermore, research on *S. schweitzeri* remains sparse, whereas current knowledge on *S. roterodami* and *S. singaporensis* is mostly limited to a single study.

The data regarding the clinical impact of *S. argenteus* also remain limited, with possible increased or decreased mortality in comparison to *S. aureus* reported in Taiwan and Australia, respectively. The regional distribution of sequence types and varying implementation of *S. argenteus* identification methods may contribute to these differences. Additionally, although no virulence factors specific to *S. argenteus* have been clearly described, *de novo* efforts to characterize these isolates (instead of comparison to known characteristics of *S. aureus*) are also needed. Many case reports to date have focused on the identification of newer SAC members and less on their clinical impact. As identification becomes more readily available, including through expanding MALDI-TOF MS databases and the increasing affordability of bacterial whole genome sequencing, future studies may be more powered to assess the clinical impact of different SAC members.

Differentiating SAC members is also important for ongoing diagnostic microbiology and infectious disease research. The rise of commercial multiplex assays for rapid diagnostics needs to consider how non-*S*. *aureus* members may affect the sensitivity of SAC detection. Novel drug development for SAC infections should also consider the unique characteristics of each species. Staphyloxanthin biosynthesis has been suggested as a potential drug target; however, *S. argenteus* would likely be intrinsically resistant (33, 78). Epitope prediction software has been used to design a potential vaccine specifically against *S. argenteus* (120).

This review shows how ongoing advances in microbiology continue to reveal new insights even among our most prevalent and well-studied bacterial pathogens. Ongoing surveillance efforts and technological advances will lead to further improvements in diagnostic microbiology, infection prevention strategies, and clinical treatment outcomes related to the *S. aureus* complex.
